# Attenuation of murine sclerodermatous models by the selective S1P_1_ receptor modulator cenerimod

**DOI:** 10.1038/s41598-018-37074-9

**Published:** 2019-01-24

**Authors:** Miyu Kano, Tadahiro Kobayashi, Mutsumi Date, Momoko Tennichi, Yasuhito Hamaguchi, Daniel S Strasser, Kazuhiko Takehara, Takashi Matsushita

**Affiliations:** 1 Department of Dermatology, Faculty of Medicine, Institute of Medical, Pharmaceutical and Health Sciences, Kanazawa University, Kanazawa, 920-8641 Japan; 2 Idorsia Pharmaceuticals Ltd., Drug Discovery, Hegenheimermattweg 91, CH-4123 Allschwil, Switzerland

## Abstract

Sphingosine-1-phosphate (S1P), a lipid mediator, regulates lymphocyte migration between lymphoid tissue and blood. Furthermore, S1P participates in several physiological phenomena including angiogenesis, inflammation, immune regulation, and neurotransmitter release. Moreover, S1P/S1P receptor signaling involves in systemic sclerosis (SSc) pathogenesis. This study aimed to investigate whether the selective S1P_1_ receptor modulator cenerimod attenuates murine sclerodermatous models. Cenerimod was orally administered to murine sclerodermatous chronic graft versus host disease (Scl-cGVHD) mice, either from day 0 to 42 or day 22 to 42 after bone marrow transplantation. Bleomycin-induced SSc model mice were administered cenerimod from day 0 to 28. Early cenerimod administration inhibited, and delayed cenerimod administration attenuated skin and lung fibrosis in Scl-cGVHD mice. Cenerimod suppressed the infiltration of CD4^+^ T cells, CD8^+^ T cells, and CD11b^+^ cells into the inflamed skin of Scl-cGVHD mice as opposed to control mice. In contrast, cenerimod increased the frequency of regulatory T cells in the spleen and skin of Scl-cGVHD mice. Additionally, cenerimod attenuated the mRNA expression of extracellular matrix and fibrogenic cytokines in the skin. Furthermore, cenerimod attenuated bleomycin-induced fibrosis in the skin and lung. Hence, the selective S1P_1_ receptor modulator cenerimod is a promising candidate for treating patients with SSc and Scl-cGVHD.

## Introduction

Systemic sclerosis (SSc) is a fibrotic disease characterized by immunologic abnormalities, vascular injury, and increased accumulation of extracellular matrix (ECM) proteins in the skin^1,2^. Effective treatment for SSc has not been established and hence warrants further investigation. Pathological analysis of SSc patients has indicated abnormal in B cell activation and differentiation^3,4^. In addition, abnormalities in T cell-derived cytokines have been reported in SSc patients^5,6^. Thus, regulation of lymphocytes is essential to treat SSc. Murine sclerodermatous chronic graft-versus-host disease (Scl-cGVHD) is a well-established model for human Scl-cGVHD and human SSc^7–9^. It can be induced by bone marrow (BM) and splenocyte transplantation from B10.D2 mice (major histocompatibility complex haplotype H-2^d^) into sublethally irradiated BALB/c (H-2^d^) mice across minor histocompatibility loci; this recapitulates the prominent characteristics of human SSc^10^.

Sphingosine-1-phosphate (S1P) is a plasma lipid mediator, which regulates various physiological phenomena including angiogenesis, inflammation, immunity, cell motility, and neurotransmitter release^11–13^. S1P_1_ receptor mediates lymphocyte egrees from thymus or secondary lymphoid tissue to blood^14,15^. FTY720 (fingolimod), a non-selective S1P_1, 2, 3, 4, 5_ receptor modulator, is an approved therapy to treat multiple sclerosis. The S1P receptor modulators are believed to exert their immune-modulating function by decreasing blood lymphocytes^16^. However, immune regulation by S1P is involved in the regulation of not only lymphocyte circulation but also lymphocyte differentiation. Therefore, S1P receptor modulaltors are an ideal therapeutic agent for various autoimmune diseases.

S1P/S1P receptor signaling reportedly plays an important role in SSc pathogenesis. Serum S1P levels are higher in SSc patients than in heathy subjects^17^. S1P_1_ and S1P_2_ receptors are reportedly downregulated and S1P_3_ receptors are upregulated in SSc dermal fibroblasts^18^. S1P promotes the fibroblast migration towards fibronectin via S1P_2_ receptors^19^. There are numerous interactions between S1P and TGF-β signaling^20^ and S1P activates the TGF-β Smad signaling pathway^21^. FTY720 reportedly ameliorates murine Scl-cGVHD and the FTY720-induced immunosuppression is believed to be primarily mediated by S1P_1_^22^. FTY720 binds not only to the S1P_1_ receptor but also to the S1P_2, 3, 4, 5_ receptors, and adverse reactions such as bradycardia are considered to occur owing to its binding to the S1P_2, 3, 4, 5_ receptors. Thus, selective S1P_1_ receptor modulators may be potent therapeutic agents to treat SSc with fewer side effects.

Cenerimod is a potent, selective, safe and orally administrable S1P_1_ receptor modulator, which reportedly reduced blood lymphocytes and attenuated murine experimental autoimmune encephalomyelitis (EAE) in a murine model^23,24^. This study aimed to investigate the effect of the selective S1P_1_ receptor modulator cenerimod on murine Scl-cGVHD model and bleomycin-induced scleroderma model.

## Results

### Cenerimod attenuates fibrosis in Scl-cGVHD mice

**Figure 1:**
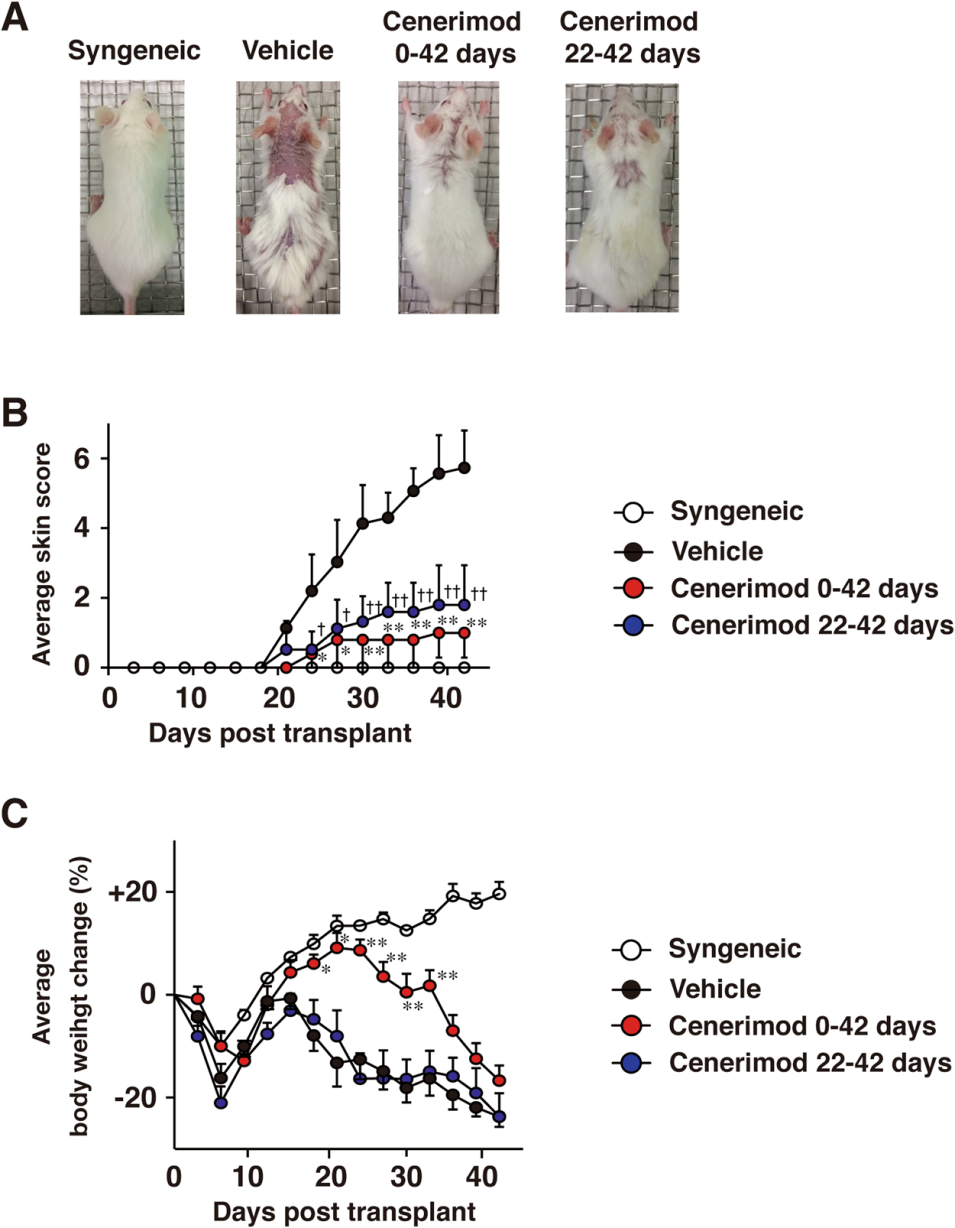
Oral treatment of cenerimod attenuates Scl-cGVHD severity and fibrosis. Recipients were given vehicle (0.25% methylcellulose, 0.05% Tween 80 in water) or were orally administered cenerimod (10 mg/kg/day) from day 0 to day 42, or from day 22 to day 42. (**A**) The respective photographs were taken 42 days after BMT. Skin score (**B**) and body weight change (**C**) were monitored every 3 days (n = 5–6 per group; *p < 0.05, **p < 0.01 for vehicle vs cenerimod day 0 to day 42. ^†^p < 0.05, ^††^p < 0.01 for vehicle vs cenerimod day 22 to day 42). All data are representative of two independent experiments.

**Figure 2:**
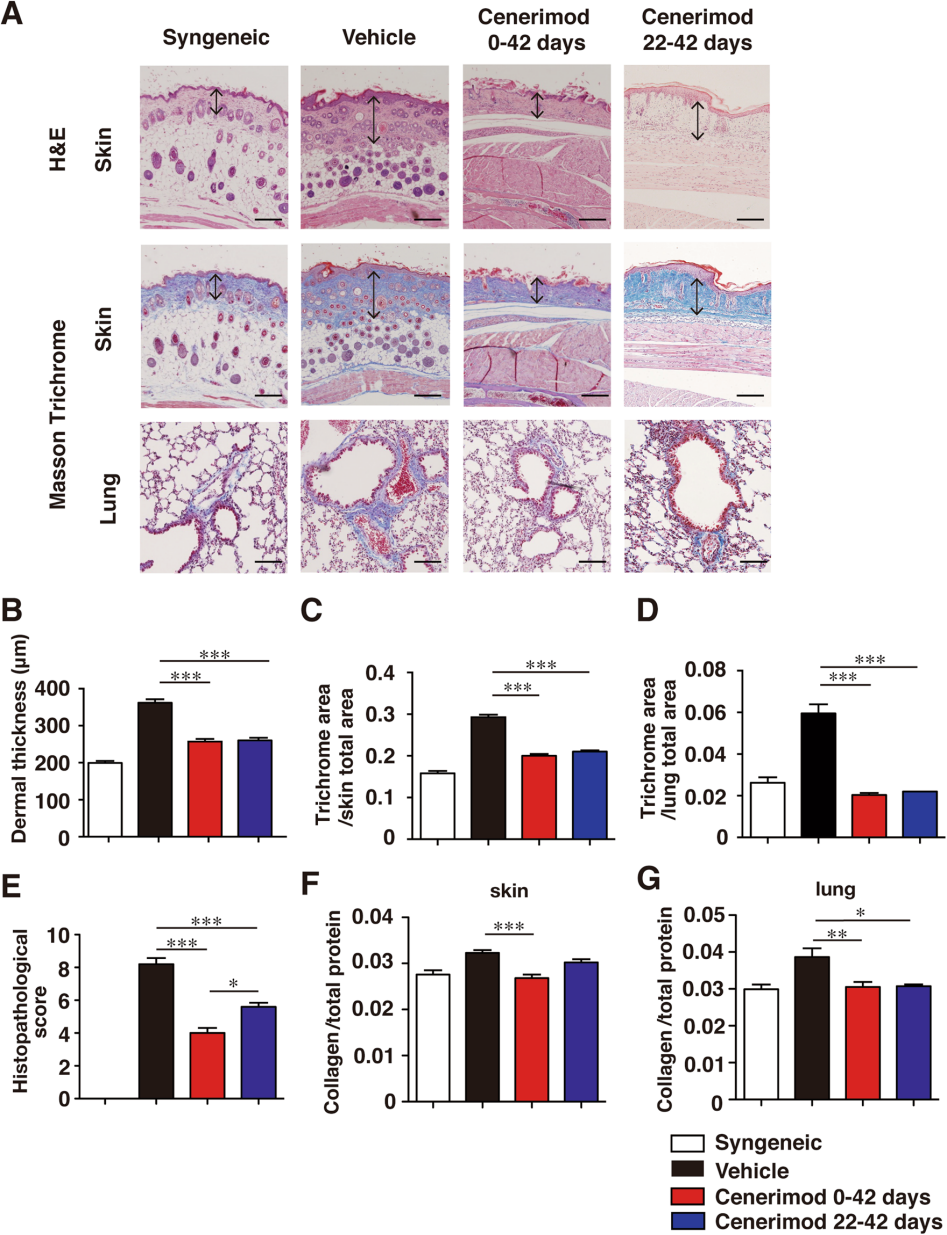
(**A**) Shown are representative photographs of histopathological changes of skin and lung at day 42 after BMT. Tissues sections are stained by Hematoxylin and Eosin (H&E) or Masson’s trichrome. Arrows indicate dermal thickness. Scale bar, 200 μm. (**B**) Dermal thickness was determined by H&E staining at day 42 after BMT. (**C**,**D**) Skin and lung fibrosis are determined as the collagen area stained by Masson’s trichrome at day 42 after BMT. The blue-stained area, representing collagen, was quantified using ImageJ software. (**E**) Histopathological score was analyzed at day 42 after BMT. (**F**,**G**) Collagen content of skin and lung at day 42 after BMT are shown (n = 4–6 mice per group; *p < 0.05, **p < 0.01, ***p < 0.001). All data are representative of two independent experiments.

To investigate whether blockade of S1P_1_ signaling regulates fibrosis, we orally administered cenerimod, a selective S1P_1_ receptor modulator, to Scl-cGVHD mice. Cenerimod treatment in both the preventive model (day 0 to day 42) and the therapeutic model (day 22 to day 42) significantly improved alopecia (Fig. 1A) and skin scores compared with the vehicle-treated (control) group (p < 0.01, Fig. 1B). The cenerimod preventive model significantly improved body weight loss, especially from day 18 to day 33, compared with the control group (day18, 21: p < 0.05, day24, 27, 30, 33: p < 0.01, Fig. 1C); however, there was no recovery in body weight loss in the preventive model from day 36 to day 42 compared with the control group. There was no significant difference in body weight loss between cenerimod therapeutic model and the control group. On histopathological analysis, dermal thickness, histopathologic score, trichrome area, and collagen content of the skin were significantly lower in cenerimod-treated groups than in the control group (p < 0.001, Fig. 2A–C,E,F). Furthermore, the fibrotic area and collagen content in the lung were significantly lower in cenerimod-treated groups than in the control group (lung collagen content vehicle vs cenerimod therapeutic model: p < 0.05, lung collagen content vehicle vs cenerimod preventive model: p < 0.01, trichrome area of lung: p < 0.001, Fig. 2D,G). Therefore, cenerimod attenuates skin and lung fibrosis in Scl-cGVHD.

### Microarray data suggest that cenerimod regulates inflammatory cytokines and Treg cells

**Figure 3:**
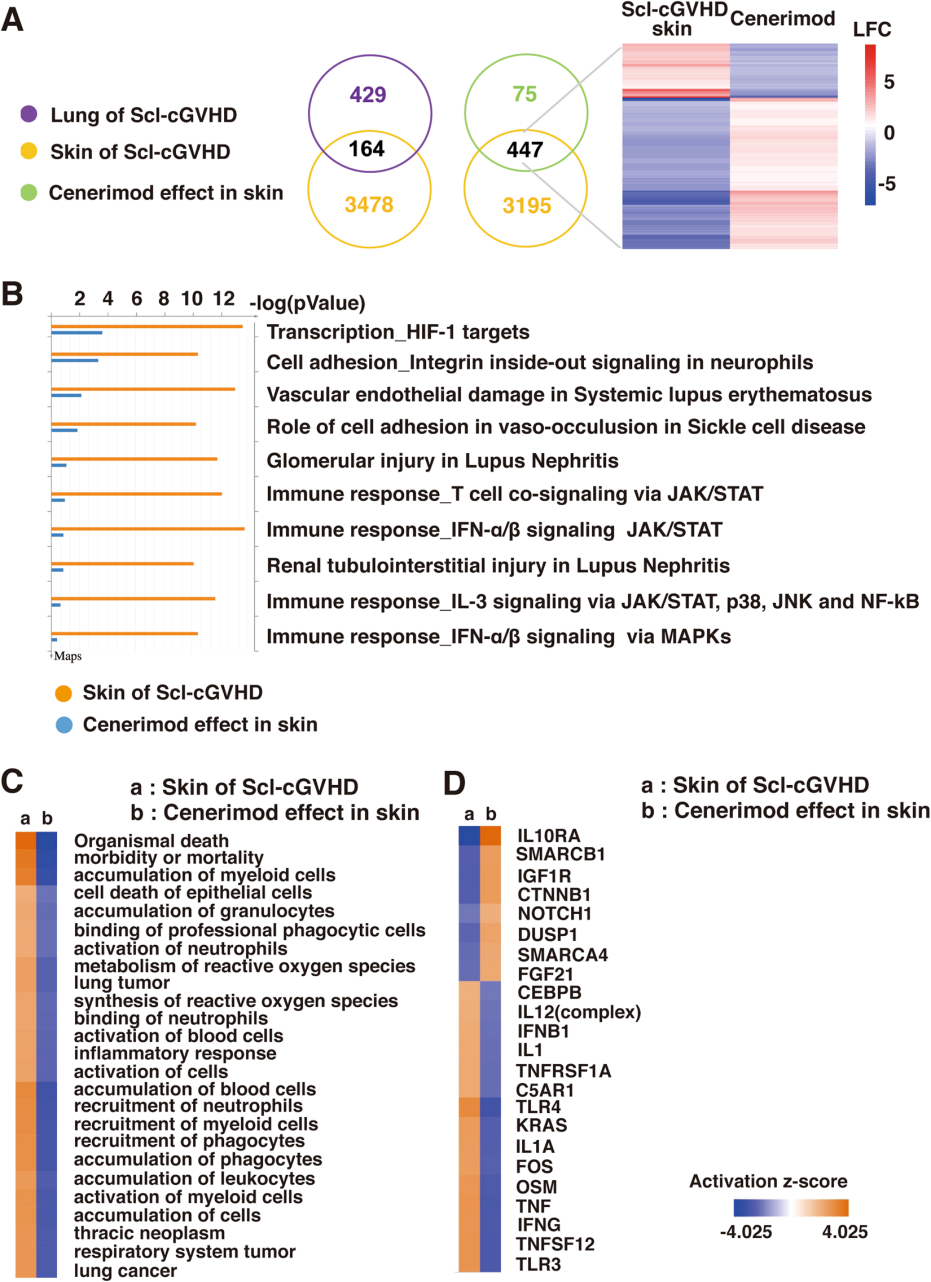
(**A**) Scl-cGVHD disease and cenerimod effect on gene expression in skin and lung. In the skin of the Scl-cGVHD mouse model, 3642 DEP were identified (FDR ≤ 0.01, LFC ≥ 1). The disease effect in the lung was weaker with 593 DEP. Cenerimod revealed a strong effect on gene expression with 522 DEP (FDR ≤ 0.01, LFC ≥ 1). Cenerimod inversely regulated 447 DEP of the Scl-cGVHD skin disease (FDR: false discovery rate, LFC: log-fold change, DEP: differentially expressed probes) (**B**) Pathway analysis of the gene expression effects using MetaCore^TM^. Both for Scl-cGVHD skin and cenerimod the major affected pathways were involved in immunological and inflammatory processes. (**C**) Pathway analysis of the gene expression effects using Ingenuity Pathway Analysis. Both for Scl-cGVHD skin and cenerimod the major affected pathways were recruitment and accumulation of immune cells such as leukocytes and granulocytes. (**D**) Pathway analysis of the gene expression effect using Ingenuity Pathway Analysis. Prediction of upstream regulators reveals a strong TNF-α, INF-γ and TLR driven disease process which is blocked by cenerimod. Conversely, IL10RA in the Scl-cGVHD mouse model was down regulated and was normalized by cenerimod. All data are representative of two independent experiments.

Microarray analysis was performed to determine the effects of cenerimod on the Scl-cGVHD model. Differentially expressed probes (DEP) were more numerous in the skin of the Scl-cGVHD than in the lung, thereby indicating a robust effect on the skin of the Scl-cGVHD mice. Additionally, cenerimod counter-regulated 477 DEP in the skin of Scl-cGVHD mice (Fig. 3A). Pathway analysis via Metacore^TM^ and Ingenuity Pathway analysis revealed interesting effects of cenerimod on cell adhesion and migration, pathways linked to systemic lupus erythematosus and lupus nephritis, and IFN-α or IFN-β signaling (Fig. 3B,C). In addition, Ingenuity Pathway analysis revealed that cenerimod demonstrated a potent counter-modulation of key genes representing TNF-α, INF-γ, and TLR4 activity (Fig. 3D). Furthermore, down-regulation of IL10RA in the Scl-cGVHD mouse model was normalized by cenerimod (Fig. 3D). Correlation Engine analysis revealed a positive correlation between Scl-cGVHD skin disease and the Scl-cGVHD regulatory T cells (Treg cells) data set (Supplementary Fig. 1A). These results suggest that cenerimod counter-regulates the effect of Scl-cGVHD on Treg cells, evident from the negative correlation (Supplementary Fig. 1B).

### Cenerimod treatment reduces immune cell infiltration into the skin

**Figure 4:**
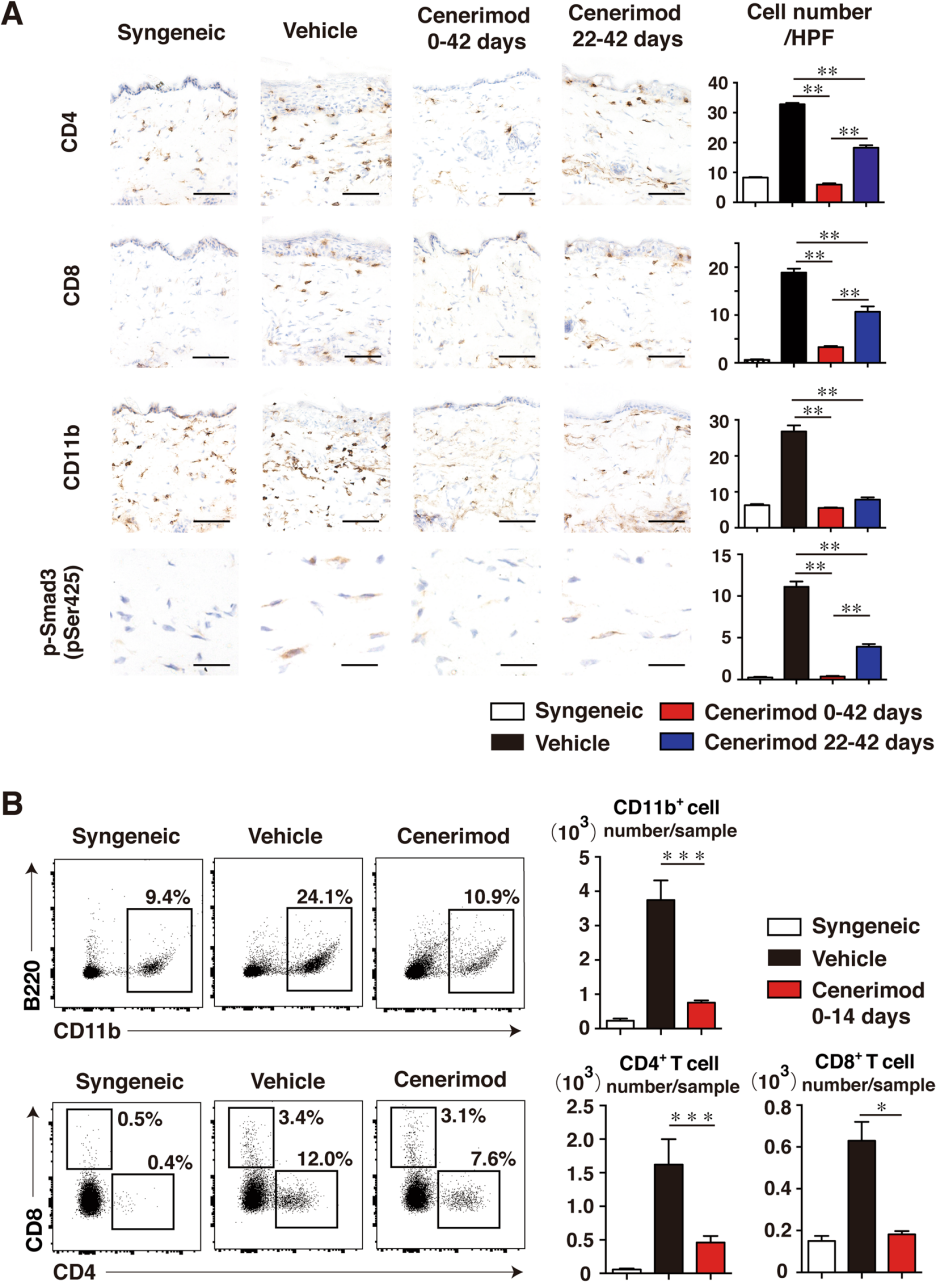
Cenerimod suppressed the infiltration of immune cells into the skin on Scl-cGVHD mice. (**A**) Shown are representative immunohistochemical staining of the skin. Skin tissues were harvested 42 days after BMT. The number of CD4^+^ T cells, CD8^+^ T cells, CD11b^+^ cells and cells containing phosphorylated Smad3 (pSer425) per high-power field (HPF) in immunohistochemical-stained slides from groups of syngeneic mice, vehicle-treated mice, cenerimod-treated mice from day 0 to day 42, and from day 22 to day 42 after BMT (n = 4–5 mice per group; **p < 0.01). Scale bar for CD4^+^ T cells, CD8^+^ T cells, CD11b^+^ cells, 50 μm. Scale bar for p-Smad3 cells, 20 μm. (**B**) Representative results demonstrate the frequency of skin-infiltrating CD11b^+^ cells, CD4^+^ T cells, and CD8^+^ T cells within indicated gates among total skin live cells. Number of skin CD11b^+^ cells, CD4^+^ T cells and CD8^+^ T cells 14 days after BMT are also shown. Values are the mean ± SEM of 4–5 mice per group (*p < 0.05, ***p < 0.001). All data are representative of two independent experiments.

To evaluate immune cell infiltration into the skin with murine Scl-cGVHD, the inflamed dorsal skin samples obtained 42 days after BMT were immunostained with anti-CD4, anti-CD8, anti-CD11b, anti-B220 and anti-phosphorylated Smad3 mAbs. Immunohistochemical staining revealed that infiltration of CD4^+^ T cells, CD8^+^ T cells, and CD11b^+^ monocyte/macrophages into the skin were significantly reduced in the cenerimod-treated group (both preventive and therapeutic model) compared with the control group (p < 0.01, Fig. 4A). In addition, preventive treatment inhibited the infiltration of CD4^+^ T cells and CD8^+^ T cells into the skin more potently than therapeutic treatment (p < 0.01, Fig. 4A). However, B220-positive cell infiltration was only observed at low levels in either groups, with no significant difference between them (data not shown). Smad3 phosphorylation was significantly higher in the skin in the allogeneic group than in the syngeneic group 42 days after BMT. Moreover, both of preventive and theraputic cenerimod treatment significantly decreased Smad3 phosphorylation in comparison with that in the control group (p < 0.01, Fig. 4A). FACS analysis also revealed that infiltration of CD4^+^ T cells, CD8^+^ T cells, and CD11b^+^ monocyte/macrophages into the skin were significantly reduced in the cenerimod-treated group 14 days after BMT compared with the control group (CD8^+^ T cells: p < 0.05, CD4^+^ T cells, CD11b^+^ cells: p < 0.001, Fig. 4B). Therefore, the modulation of S1P_1_ receptor significantly decreased immune cell infiltration into the skin of Scl-cGVHD mice.

### Cenerimod increases B cells and regulatory T cells

**Figure 5:**
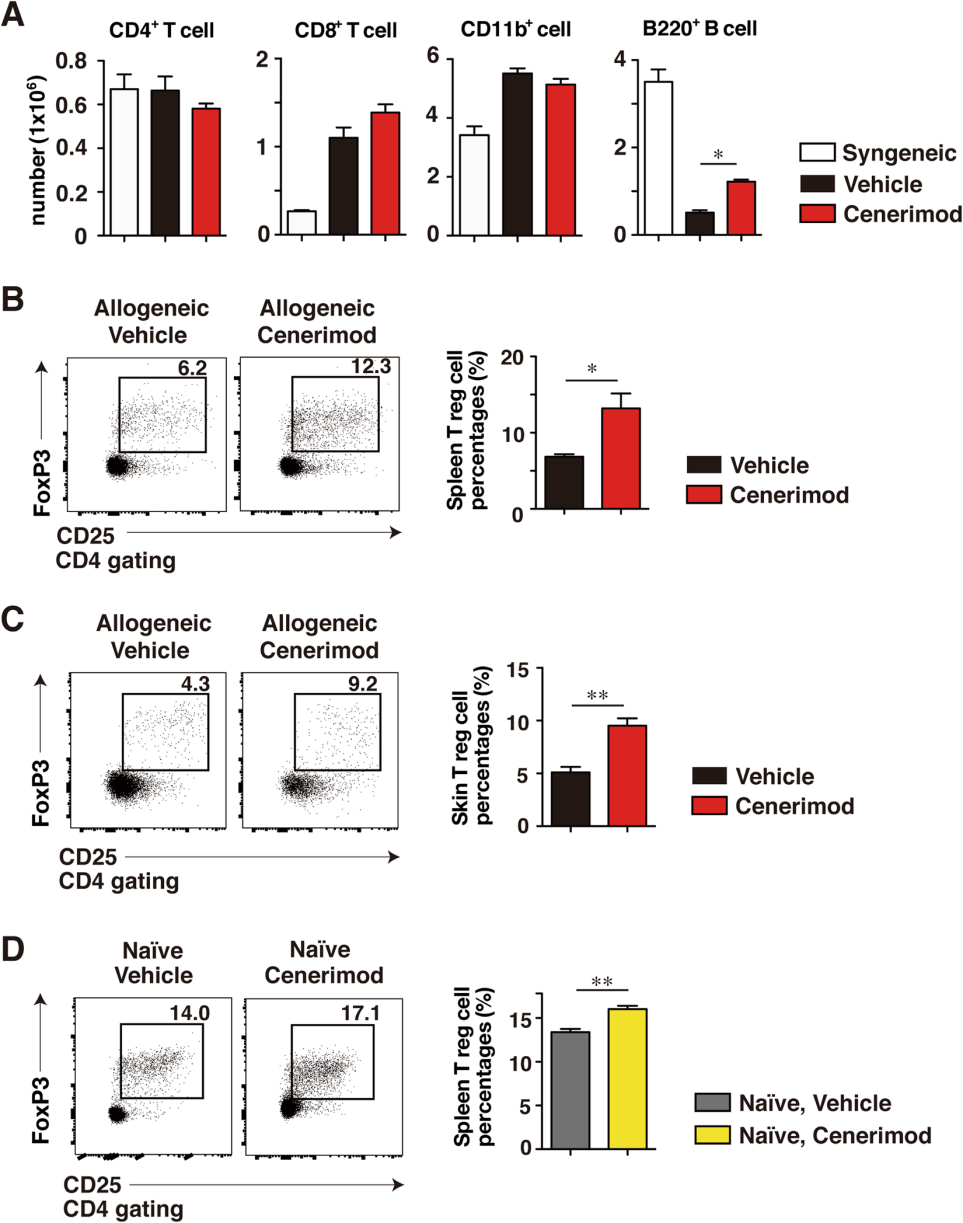
(**A**) Shown are the number of splenic CD4^+^ T cells, CD8^+^ T cells, CD11b^+^ cells and B220^+^ B cells 14 days after BMT enumerated via FACS analysis. Values are the mean ± SEM of 3–5 mice per group (*p < 0.05). (**B**) Representative results demonstrate the frequency of splenic CD25^+^ FoxP3^+^ T cells in allogeneic BMT mice within indicated gates among total CD4^+^ spleen live cells. The percentages of splenic CD25^+^FoxP3^+^ T cells within splenic CD4^+^ cells 14 days after BMT are also shown (n = 4–5 mice per group; *p < 0.05). (**C**) Representative results demonstrate the frequency of skin CD25^+^ FoxP3^+^ T cells in allogeneic BMT mice within indicated gates among total CD4^+^ skin live cells. The percentages of skin CD25^+^FoxP3^+^ T cells within skin CD4^+^ cells 14 days after BMT are also shown (n = 4–5 mice per group; **p < 0.01). (**D**) Representative results demonstrate the frequency of splenic CD25^+^ FoxP3^+^ T cells in BALB/C mice within indicated gates among total CD4^+^ spleen live cells. The percentages of splenic CD25^+^FoxP3^+^ T cells within splenic CD4^+^ cells at day 14 are also shown (n = 4–5 mice per group; **p < 0.01). All data are representative of two independent experiments.

We enumerated the CD4^+^ T cells, CD8 T^+^ cells, CD11b^+^ cells, and B220^+^ B cells in the spleen 14 days after BMT. The number of B220^+^ B cells in the spleen was significantly higher in the cenerimod-treated group than in the control group (p < 0.05, Fig. 5A), while there was no significant difference in the number of CD4^+^ T cells, CD8 T^+^ cells, and CD11b^+^ cells between cenerimod-treated group and the control group (Fig. 5A). We enumerated Treg cells (CD4^+^CD25^+^FoxP3^+^ cells) in the spleen and skin of allogeneic BMT mice 14 days after BMT. The percentage of Treg cells both in the spleen and skin was significantly higher in the cenerimod-treated group than in the control group (spleen: p < 0.05, skin: p < 0.01, Fig. 5B,C). Furthermore, cenerimod also increased the percentage of splenic Treg cells in the naïve BALB/c mice compared with BALB/c mice administered with vehicle (p < 0.01, Fig. 5D). Thus, cenerimod treatment increased the Treg cells in not only Scl-cGVHD mice but also naïve mice.

### Cenerimod downregulates cytokine mRNAs in the skin of Scl-cGVHD mice

**Figure 6:**
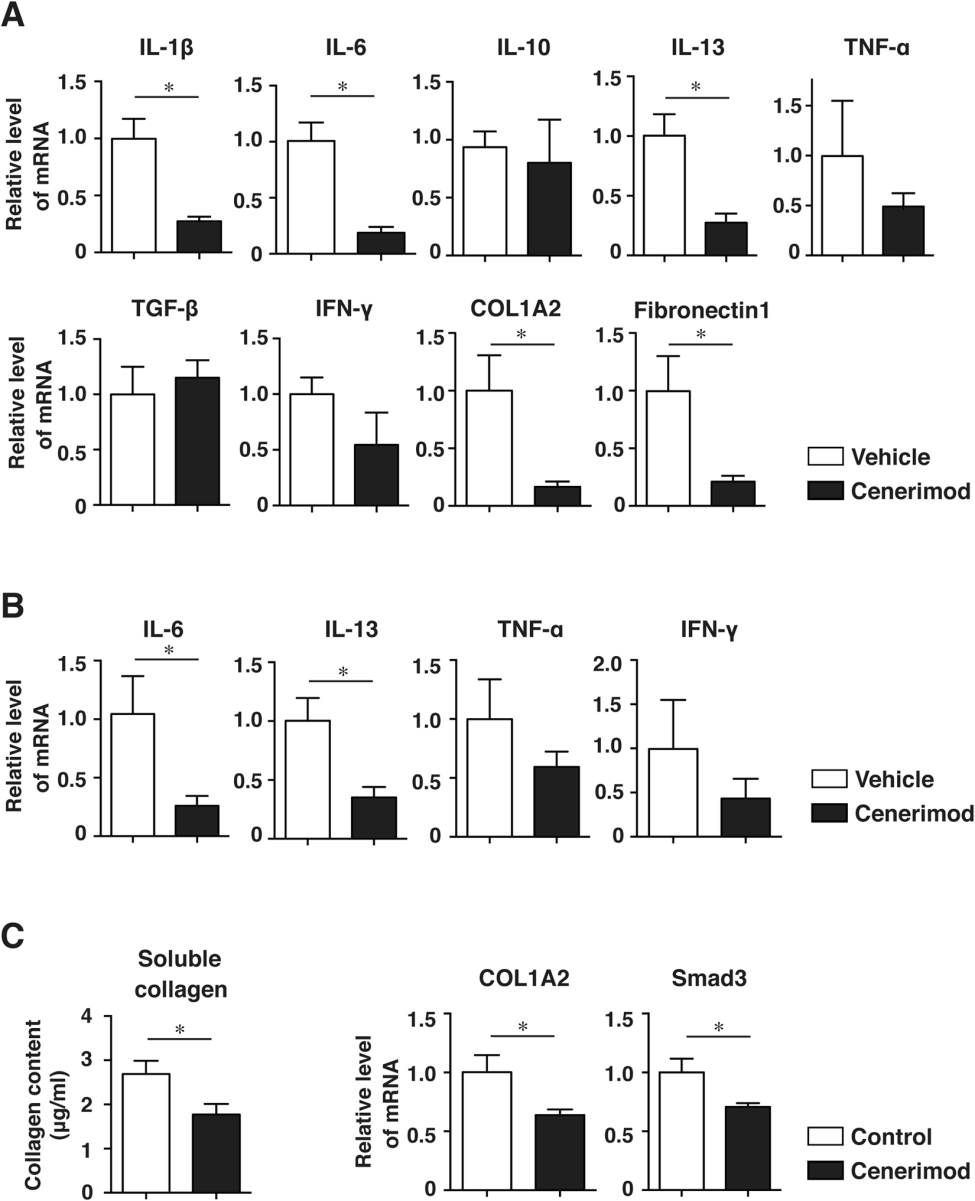
(**A**) Expression of mRNA for interleukin-1β (IL-1β), IL-6, IL-10, IL-13, tumor necrosis factor α (TNF-α), transforming growth factor β (TGF-β), interferon-γ (IFN-γ), and proα2 (I) collagen (COL1A2) and fibronectin 1 in the skin of allogeneic groups were measured by real-time quantitative polymerase chain reaction (RT-PCR) analysis 42 days after BMT (n = 4–6 mice per group; *p < 0.05). (**B**) Skin-infiltrating cells of allogeneic BMT mice were T-cell-selected with anti-Thy1.2 microbeads. Expression levels of IL-6, IL-13, TNF-α, and IFN-γ mRNA were measured in the skin-infiltrating T cells via real-time RT-PCR analysis 14 days after BMT (n = 4–5 mice per group; *p < 0.05). (**C**) Cultured fibroblasts were pretreated with vehicle or cenerimod (5 μmol/L) and incubated with TGF-β2 (10 ng/mL) for 24 h. The collagen content of fibroblast supernatants was determined using a Quickzyme Soluble Collagen Assay kit. COL1A2 and Smad3 mRNA expression in cultured fibroblasts were quantified via real-time RT-PCR analysis. Values are presented as mean ± SEM values of 4–5 experiments per group (*p < 0.05). All data represent two independent experiments.

Expression of cytokines, type I collagen gene proα2 (COL1A2) and fibronectin 1 mRNA was assessed in the skin of Scl-cGVHD mice via real-time PCR analysis (Fig. 6A). Fibrogenic cytokines including IL-1β, IL-6, and IL-13 mRNA were significantly downregulated in the cenerimod-treated group compared to the control group (p < 0.05, Fig. 6A). TNF-α and IFN-γ mRNA expression were slightly but not significantly lower in the cenerimod-treated group than in the control group. IL-10 and TGF-β mRNA expression levels did not change with cenerimod treatment. COL1A2 and fibronectin 1 mRNA were also significantly downregulated in the skin of cenerimod-treated mice compared with the control mice (p < 0.05, Fig. 6A). Together, cenerimod treatment downregulated mRNA of fibrogenic cytokines and ECM proteins. To assess cytokine production in skin T cells, we isolated T cells from the skin of Scl-cGVHD mice. Cenerimod treatment significantly downregulated IL-6 and IL-13 mRNA in the skin T cells (p < 0.05, Fig. 6B). TNF-α and IFN-γ mRNA expression in the cenerimod-treated group tended to be slightly but not significantly downregulated in comparison with that in the control group (Fig. 6B).

### Cenerimod inhibits collagen production in fibroblasts

To directly investigate the effects of cenerimod on fibroblasts, skin fibroblasts were cultured and treated with cenerimod. Consequently, soluble collagen content of fibroblast culture supernatant decreased after cenerimod treatment (p < 0.05, Fig. 6C). Furthermore, COL1A2 and Smad3 mRNA were significantly downregulated in the cenerimod-treated group compared to that in the vehicle treatment group (p < 0.05, Fig. 6C). Therefore, collagen production of fibroblasts was suppressed by S1P_1_ modulation.

### Cenerimod attenuates fibrosis in the bleomycin-induced scleroderma model

**Figure 7:**
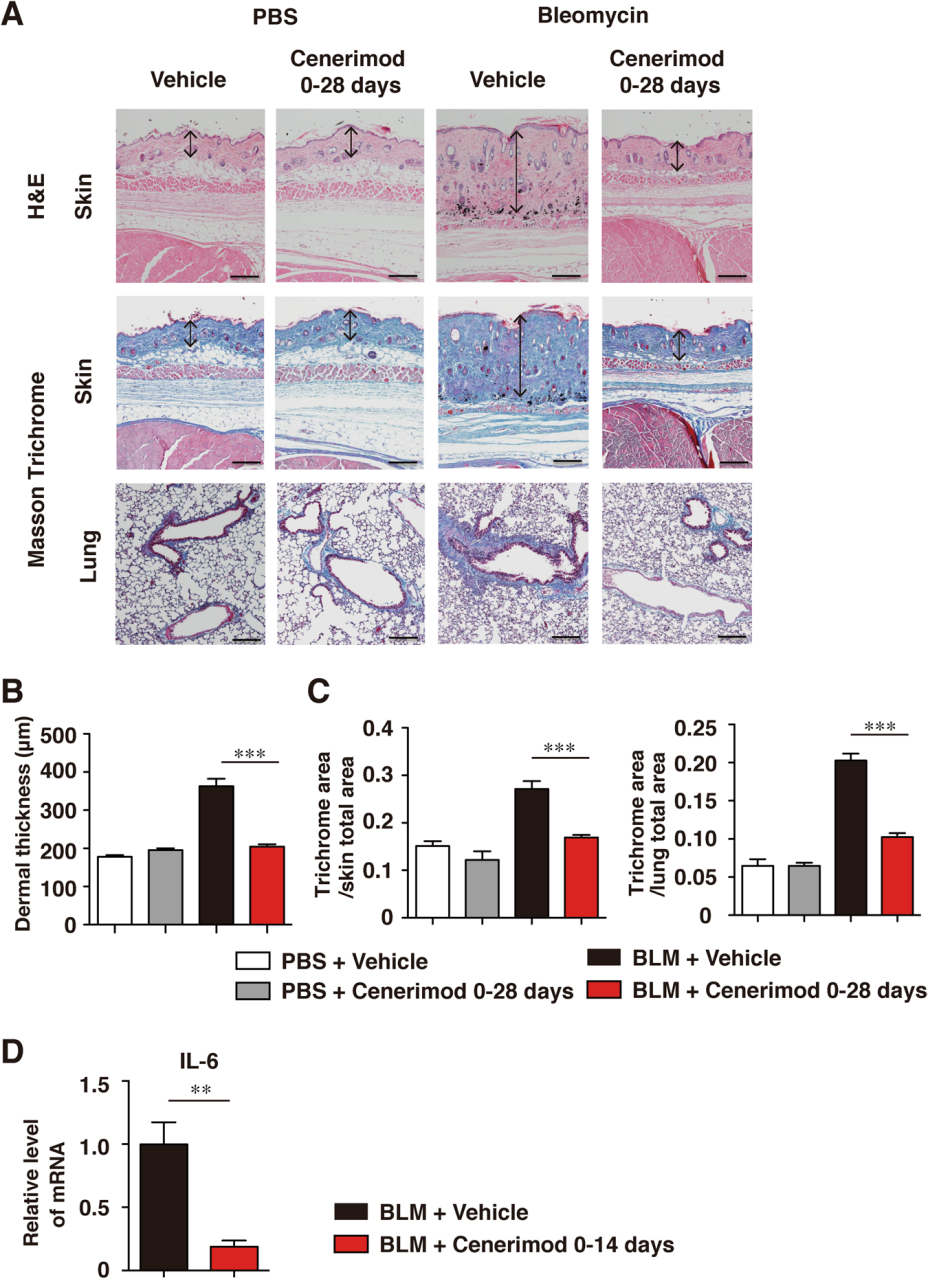
Skin fibrosis induced by intradermal bleomycin injections. (**A**) Shown are representative photographs of histopathological changes of skin and lung at day 28. Tissues sections are stained by Hematoxylin and Eosin (H&E) or Masson’s trichrome. Arrows indicate dermal thickness. Scale bar, 200 μm. (**B**) Dermal thickness was determined by H&E staining at day 28. (**C**) Skin and lung fibrosis are determined as the collagen area stained by Masson’s trichrome at day 28. The blue-stained area, representing collagen, was quantified using ImageJ software (n = 7–9 mice per group; ***p < 0.001). (**D**) IL-6 mRNA expression in the skin was quantified via real-time RT-PCR analysis 14 days after bleomycin treatment (n = 4–6 mice per group; *p < 0.05). All data are representative of two independent experiments.

To investigate whether cenerimod attenuates fibrosis in an another mouse model of SSc, we used a bleomycin-induced scleroderma model induced via intradermal administration of bleomycin, which then developed skin and lung fibrosis^25^. Histopathological analysis revealed that dermal thickness was significantly lesser in the cenerimod-treated group than in the control group (p < 0.001, Fig. 7A,B). Furthermore, the fibrosis area in the skin and lung were significantly lesser in cenerimod-treated group than in the control group (p < 0.001, Fig. 7C). Moreover, IL-6 mRNA level in the skin of the bleomycin model mice was significantly downregulated in the cenerimod-treated group compared to that in the control group (p < 0.05, Fig. 7D). IL-13 mRNA in the skin was also downregulated to below the measurement sensitivity upon cenerimod treatment, although IL-13 mRNA was detected in the control treatment group (data not shown). Thus, cenerimod also suppressed fibrosis in a mouse model of bleomycin-induced scleroderma.

## Discussion

This study is the first to evaluate the therapeutic effects of the selective S1P_1_ receptor modulator cenerimod on a murine Scl-cGVHD model and a bleomycin-induced scleroderma model. The orally administered cenerimod not only prevented but also attenuated the development of skin and lung fibrosis in Scl-cGVHD mice. Furthermore, the S1P_1_ receptor modulator attenuated skin and lung fibrosis in a mouse model of bleomycin-induced scleroderma. Infiltration of CD4^+^ T cells, CD8^+^ T cells, and CD11b^+^ cells into the skin were significantly lower in the cenerimod-treated group than in the control group. Additionally, cenerimod increased the number of splenic and skin Treg cells in Scl-cGVHD mice. Furthermore, cenerimod reduced the mRNA expression of fibrogenic cytokines and ECM proteins in the skin and decreased the fibrogenic cytokines in the skin T cells of Scl-cGVHD mice. In addition, collagen synthesis from fibroblasts was directly inhibited by cenerimod treatment. Hence, the selective S1P_1_ receptor modulator cenerimod is a promising therapeutic agent for human sclerodermatous chronic GVHD and SSc.

S1P/S1P receptor modulators constitute promising therapy for autoimmune diseases, since S1P receptor modulators have a strong immunosuppressive effect. FTY720, a S1P_1, 2, 3, 4, 5_ receptors modulator, is being used to treat multiple sclerosis. Furthermore, the effect of FTY720 will be canceled immediately irrespective of whether therapy is discontinued. However, FTY720 has serious side effects including bradycardia and bronchoconstriction owing to inhibition of S1P_2, 3, 4, 5_ receptors. Cenerimod is a highly selective S1P_1_ receptor modulator along with the absence of adverse events such as vasoconstriction and bronchoconstriction^24^. Thus, compared to FTY 720, cenerimod is safer and beneficial for treating autoimmune diseases. Activation of the S1P_1_ receptor by S1P causes lymphocyte outflow from lymphoid tissues into the tissue stream and accelerates lymphocyte migration. Conversely, circulating lymphocytes are decreased upon functional antagonism of the S1P_1_ receptor with cenerimod^23,24^. Donor-derived immune cell infiltration into the skin has been observed in early Scl-cGVHD mice^10^. FTY720 inhibits Scl-cGVHD via blockade of lymphocyte migration^22^. Administration of cenerimod to Scl-cGVHD mice reduced the infiltration of CD4^+^ T cells, CD8^+^ T cells, and macrophages in the skin. This finding indicates that T cell and macrophage infiltration in the skin were also reduced upon selective S1P_1_ receptor modulation. Hence, cenerimod exerts immunosuppressive effects by reducing T cells and macrophages invading the immune reaction site, since the circulating lymphocytes are suppressed.

Cytokines have essential functions in fibrosis in SSc pathogenesis^5,26,27^. Based on previous results, cytokine balance is thought to be inclined towards Th2 in SSc^28,29^. Th2 cytokines, such as IL-4, IL-6, and IL-13 stimulate collagen production in human fibroblasts *in vitro* and play an essential role in SSc fibrosis^8,30–33^. Cenerimod downregulated fibrogenic Th2 cytokine and collagen mRNA in the skin of Scl-cGVHD mice. The present findings indicate that collagen production from fibroblasts was suppressed by decreasing these Th2 cytokines. Furthermore, cenerimod altered T cell phenotype in the skin. These results indicate that cytokine reduction in the skin is due to decreased T cell and macrophage infiltration and alteration of T cell function. Additionally, collagen production from fibroblasts was significantly inhibited by cenerimod *in vitro*. Although TGF-β mRNA expression in the skin was not changed with cenerimod treatment, cenerimod treatment significantly attenuated Smad3 phosphorylation in the skin and Smad3 mRNA expression of fibroblast *in vitro*. Thus, the current study suggests that cenerimod directly inhibited collagen production from fibroblasts through down-regulation of TGF-β-Smad signaling pathway. Together, attenuation of fibrosis is not only due to the reduction in Th2 cytokines, but also due to the direct inhibition of fibroblasts by cenerimod.

Treg cells suppress T cell activation, and essentially maintain immune tolerance and suppression of autoimmune development^34^. The impairment of Treg cell function is associated with the development of autoimmune diseases^35^. Treg frequency is increased in early SSc^36–38^ and decreased in late SSc^39–41^. Furthermore, the imbalance of Treg cells and effector T cells accelerate the pathogenesis of SSc^38,42,43^. Additionally, Treg cells are an important factor in suppressing GVHD. The beneficial effects of treating GVHD mice with FTY720 or other therapeutic agents depends on an increase in the number of Treg cells^44,45^. In the current study, microarray data revealed that cenerimod activity is inversely correlated with the modulation of the effects of Treg cells in Scl-cGVHD. Treg cell number and function are enhanced in S1P_1_ receptor-deficient mice and conversely, Treg cell function is attenuated in S1P_1_ receptor transgenic mice^46^. Moduation of S1P_1_ receptors by FTY720 enhances Treg cell number and function^46,47^. Cenerimod also increases the percentage of Treg cells both in the spleen and skin. Collectively, the beneficial effect of cenerimod also depends on an increase in the splenic and skin Treg cells in a mouse model of Scl-cGVHD.

In summary, this study shows that the selective S1P_1_ receptor modulator cenerimod attenuates skin and lung fibrosis in a mouse model of Scl-cGVHD and that of bleomycin-induced scleroderma. Cenerimod apparently exerts an immunosuppressive effect with attenuation of immune cell infiltration into the skin, decreased fibrogenic cytokine environment, accompanied by an increase in splenic and skin Treg cell number. The present results indicate that the selective S1P_1_ receptor modulator cenerimod can be a promising candidate to treat human Scl-cGVHD and SSc.

## Materials and Methods

### Mice

B10.D2 (H-2^d^), BALB/c (H-2^d^) and C57BL/6 mice were purchased from Japan SLC (Shizuoka, Japan). The mice were housed in a specific pathogen-free barrier facility. All studies and procedures were approved by the Committee on Animal Experimentation of Kanazawa University Graduate School of Medical Science. All mouse experiments were conducted based on ethical guidelines of Kanazawa University.

### Bone marrow transplantation

8–12-week-old male B10.D2 and female BALB/c mice were used as donors and recipients, respectively. Bone Marrow (BM) was T cell-depleted (TCD) with anti-Thy1.2 microbeads (Miltenyi Biotec, Auburn, CA). BALB/c recipients were irradiated with 400 cGy twice a day (MBR-1520R; Hitachi, Tokyo, Japan) at one day before transplantation and were injected via the tail vein with 10 × 10^6^ TCD-BM and 10 × 10^6^ splenocytes in 0.5 mL phosphate-buffered saline (PBS) to generate Scl-cGVHD (allogeneic BMT). A control syngeneic group of female BALB/c mice received male BALB/c TCD-BM and splenocytes (syngeneic BMT) as we described previously in our study^22^.

### Intradermal bleomycin treatment

Bleomycin was dissolved in PBS at a concentration of 1 mg/ml. C57BL/6 mice received intradermal injections of either bleomycin or PBS (300 μl, administered using a 27-gauge needle) into their shaved backs every other day for 4 weeks, as described previously^25^.

### Reagents

Cenerimod was provided by Idorsia Pharmaceuticals Ltd.^24^ It was orally administered to allogenic recipients or bleomycin injected mice at a dose of 10 mg/kg/day. Scl-cGVHD mice were administered cenerimod from day 0 to day 42 (preventive model) or day 22 to day 42 (therapeutic model) after BMT. Bleomycin injected mice were administered cenerimod from day 0 to day 28. Control group mice received vehicle. The vehicle was 0.25% methylcellulose (Sigma-Aldrich, St. Louis, MO) and 0.05% Tween 80 (Sigma-Aldrich) in water.

### GVHD skin score

Mice were weighed every 3 days after BMT and scored for skin lesion as previously described^48^. The scoring was as follows: healthy appearance, 0; skin lesions with alopecia equal to or less than 1 cm^2^ in area = 1; skin lesions with alopecia 1 to 2 cm^2^ in area = 2; skin lesions with alopecia 2 to 5 cm^2^ in area = 3; skin lesions with alopecia 5 to 10 cm^2^ in area = 4; skin lesions with alopecia 10 to 15 cm^2^ in area = 5; skin lesions with alopecia 15 to 20 cm^2^ in area = 6; skin lesions with alopecia more than 20 cm^2^ in area = 7. The skin lesion area was traced on a paper, and the traced area was scanned and measured using the ImageJ software (http://rsb.info.nih.gov/ij). Furthermore, animals were assigned 0.4 points for skin disease (lesions or scaling) on the tail and 0.3 points each for lesions on the ears and paws. The minimum and maximum scores were 0 and 8, respectively.

### Histological analysis

The skin and lung samples were fixed in 10% formalin and embedded in paraffin. Sections (6 µm in thickness) were stained with hematoxylin and eosin (H&E) and Masson’s trichrome. Skin histopathology score was determined on the basis of epidermal interface change, dermal collagen thickness, mononuclear cell inflammation, subdermal fat loss, and follicular dropout^49^. The scores were evaluated from 0 to 2 for each category. The minimum score was 0, and the maximum score was 10. It was assessed independently by a dermatopathologist in a blinded manner (T.K. and M.T.). Dermal thickness was measured from the upper dermis to the lower dermis by microscopy. The blue-stained area on Masson’s trichrome staining, representing collagen, was quantified using ImageJ software, as we described previously in our study^22^.

### Measurement of collagen content in tissue samples

The skin and lung samples were fixed in 10% formalin and embedded in paraffin. Sections (15 µm in thickness) were deparaffinized after incubation with xylol, xylol:ethanol (1:1), ethanol, water: ethanol (1:1), and water. Individual samples were placed in small test tubes and covered with 0.2 ml of a saturated solution of picric acid in distilled water that contained 0.1% fast green FCF and 0.1% sirius red F3BA. The samples were rinsed several times with distilled water until the fluid was colorless. One milliliter of 0.1 N NaOH in absolute methanol (1:1 volume:volume) was added, and the eluted color was read on a spectrophotometer at 540 nm and 605 nm, as we described previously in our study^50^. The method used is based on the selective binding of sirius red F3BA and fast green FCF to collagen and noncollagenous protein, respectively^51^.

### Gene expression analysis and biological interpretation

The mice lung and skin tissue samples were collected in RNA later (Ambion, Carlsbad, CA). Tissues samples were lysed and homogenized in TRI Reagent (AM9738, Ambion) using the Tissue Lyzer (Qiagen, Hilden, Germany). Total RNA was isolated from 1 ml of the tissue lysate using MagMax 96 microarray kit (AM1839, Life Technologies, Carlsbad, CA) according to the manufacturer’s instructions. Quality control was performed using NanoDrop ND-1000® (NanoDrop Technologies, Wilmington, NC) and 2200 Tape Station® (Agilent Technologies, Santa clara, CA). All samples passed the quality thresholds 1.8 < 260/280 ratio <2.2 and the RNA integrity number was >7.00. Amplification and labeling were performed with Agilent Low RNA Input Quick Amp Labeling Kit (Agilent Ref. 5190-2305/2307) according to the manufacturer’s instructions using 100 ng of total RNA as starting material. The two color protocol was used, where mouse RNA reference (Agilent Stratagen) was labeled in Cy5, and all the study samples were labeled in Cy3. Different labeling of mouse RNA reference in Cy5 were performed in parallel, where all Cy5 labels passed the QC thresholds and were pooled together to constitute a single batch of cRNA Mice Ref_Cy5. The 8 × 60 K multiplex arrays with 50,599 60-mer oligonucleotides directed against the mice transcriptome were purchased from Agilent (SurePrint G3 Mice GE 8 × 60k, Ref. G4852A, design ID 028005. Each Cy3 samples (300 ng) were hybridized against the cRNA Mice Ref_Pool_Cy5 (300 ng) which was used as internal calibrator for normalization. The samples were hybridized according to the randomized design. Hybridizations and washes were performed following the manufacturer’s instructions. The arrays were scanned using the Agilent microarray scanner (Ref. G2565BA) and Scan Control software 8.5.1, data were extracted with the Feature Extraction software 11.5.1.1.

Microarray raw data were normalized using the normalize VSN function of the R/Bioconductor package vsn with minDataPointsPerStratum = 1^52^. Statistical analysis was performed using R and Limma (Smyth 2004) on the probe level with p-value adjustment using the algorithm of Benjamini and Hochberg algorithm. Probes with a log2 fold-change >=1 and a FDR <=0.01 were considered statistically significantly regulated for skin or lung. The same cutoffs were used to determine commonly regulated probes between lung and skin, in the Scl-cGVHD mouse versus syngeneic BMT mouse. To visualize relations between comparisons, Venn diagrams were generated using Limma’s vennCounts and vennDiagram functions. The lists of significantly regulated probes defined with Limma were uploaded into Ingenuity Pathway Analysis (IPA, Ingenuity System, Qiagen), BaseSpace Correlation Engine analysis (Illumina, San Diego, CA) and GeneGo Metacore (version Sep 2017).

### Immunohistochemical staining of the skin

The skin samples from Scl-cGVHD mice were removed and frozen in liquid nitrogen using embedding medium for frozen tissue specimens (Tissue-Tek OCT compound; Sakura Finetek, Tokyo, Japan) and stored at −70 °C until use. As we described previously in our study^22^, Frozen sections (5µm-thick) were immediately fixed in cold acetone and were incubated with rat anti-mouse CD4 monoclonal antibody (mAb) (RM4–5 clone; BD Biosciences, San Jose, CA), rat anti-mouse CD8 mAb (53-6.7 clone; BD Biosciences), rat anti-mouse CD11b mAb (M1/70 clone; BD Biosciences), antibody against Smad3 phosphorylated at Ser425 (NBP1-72209; Novus Biologicals, Littleton, CO). The paraffinized skin sections were applied to slides. Before B220 immunostaining, the slides were deparaffinized in xylene and 3% H_2_O_2_, hydrated through graded alcohols, and washed in distilled water. After that, the slides were put in the protease K (S3020; DAKO, Santa Clara, CA) for 20 min and were blocked by incubating the slides for 30 min in 5% skim milk with TBS. The sections were incubated with rat anti-mouse B220 mAb (RA3-6B2 clone; BD Biosciences). CD4 mAb, CD8mb, CD11b mAb and B220 mAb sections were then incubated sequentially with a biotinylated goat anti-rabbit IgG secondary antibody (BD Biosciences). The antibody against Smad3 phosphorylated at Ser425 sections were incubated sequentially with a rabbit IgG-heavy and light chain cross-absorbed secondary antibody (Bethyl Laboratories, Montgomery, TX). After those, all sections were incubated with horseradish peroxidase–conjugated avidin–biotin complex (Vectastain ABC method; Vector Laboratories, Burlingame, CA). All sections were washed 3 times with PBS between incubations, developed with 3,3ʹ-diaminobenzidine tetrahydrochloride and H_2_O_2_, and then counterstained with hematoxylin. Positive cells were counted in five high-power fields (HPF) and the average/HPF was calculated.

### Preparation of skin cell suspensions

As we described previously in our study^53^, a 1 × 3 cm piece of depilated back skin was minced and then digested in 5 mL of RPMI-10% fetal bovine serum containing 2.5 mg/mL collagenase D (Roche, Basel, Switzerland), 1.5 mg/mL hyaluronidase (Sigma-Aldrich), and 0.03 mg/mL DNase I (Roche) at 37 °C for 90 min. Digested cells were then passed through a 70-μm cell Falcon cell strainer (BD Biosciences) to generate single-cell suspensions. The cell suspension was centrifuged at 300 × *g* for 10 min. The pellet was resuspended in 70% Percoll solution (GE Healthcare, Uppsala, Sweden), and then overlaid by 37% Percoll solution followed by centrifugation at 500 × *g* for 20 min at room temperature. Cells were aspirated from the Percoll interface and passed through a 70-μm cell strainer. Subsequently, the cells were harvested by centrifugation and washed.

### Flow cytometry

The following Abs to mouse antigens were used, anti CD4-Fluorecen isothiocyanate (FITC) (RM4–5), anti CD8a-PE-Cy7 (53-6.7), anti CD11b-PerCP-Cy5.5 (M1/70), anti CD19-PE-Cy7 (6D5), anti CD25-PerCP-Cy5.5 (PC61), anti B220-APC-Cy7 (RA5-6B2), anti F4/80-BV421 (BM8) from BioLegend (San Diego, CA), anti CD4-PE (L3T4 GK1.5) from BD Biosciences, and anti FoxP3-PE (FJK-16S) from eBioscience (Waltham, MA). The Live/Dead Fixable Aqua Dead Cell Stain kit was from Molecular probes (Waltham, MA). As we described previously in our study^53^, splenic and skin single cell suspensions were stained for 20 min for 2 to 4 color immunofluorescence at 4 °C using mAbs at predetermined optimal concentrations. After finished surface staining, the cells were washed, fixed and permeabilized for 30 min at 4 °C using the Foxp3/Transcription Factor Staining Buffer Set (eBioscience). Cells were washed with Permeabilization Buffer (eBioscience), and stained for FoxP3 and incubated for 30 min at room temperature. Stained samples were analyzed using FACS CantoII (BD Biosciences). Date were analyzed using FlowJo software (Tree Star, San Carlos, CA).

### Fibroblast culture

The C57BL/6 mouse Primary Deremal Fibroblasts were purchased from Cell Biologics (Chicago, IL). As we described previously in our study^53^, fibroblasts were cultured in DMEM (Invitrogen, Carlsbad, CA) containing 10% heat-inactivated FCS, 100 U/mL penicillin (Invitrogen), and 100 µg/mL streptomycin (Invitrogen) at 37 °C in a humidified 5% CO_2_ atmosphere. Outgrowing fibroblasts were detached by brief trypsin treatment and recultured in the medium. Confluent cultures of fibroblasts were serum starved for 12 h and then pretreated with cenerimod for one hour. The cells were stimulated with 10 ng/mL TGF-β2 (BioLegend) and incubated for another 24 h. The supernatant was harvested, and the monolayers were washed. The cells were used immediately in experiments, as indicated. All experiments involved fibroblasts between passages 2 and 5, depending on the number of cells obtained initially from the tissue samples. Cultured fibroblasts adhered to the dish and maintained their typical spindle-shaped morphology. In each experiment, all cell lines were examined simultaneously under the same conditions (e.g., cell density, passage, days after plating, etc.) as we described previously^53^. The concentration of soluble collagen in cultured fibroblast supernatants was determined using the Quickzyme Soluble Collagen Assay Kit (QuickZyme Biosciences, Leiden, The Netherlands).

### Reverse transcription–polymerase chain reaction

Skin was harvested 42 days after BMT or 14 days after intradermal bleomycin treatment. The skin infiltrating cells 14 days after BMT were T cell-separated with anti-Thy1.2 microbeads (Miltenyi Biotec). Total RNA was isolated from frozen skin specimens, skin infiltrating cells and cultured fibroblasts using RNeasy spin columns (Qiagen) and digested with DNase I (Qiagen) to remove chromosomal DNA. Total RNA was reverse-transcribed to complementary DNA using a reverse transcription system with random hexamers (Promega, Southampton, UK). Cytokine messenger RNA (mRNA) was analyzed using real-time reverse transcription–polymerase chain reaction (RT-PCR) quantification (Applied Biosystems, Foster City, CA). Sequence-specific primers and probes designed and predeveloped Taqman assay reagents (Applied Biosystems) were used. The numerical descriptors are as follows; interleukin-1β (IL-1β): Mm00434228, IL-6: Mm00446190, IL-10: Mm99999062, IL-13: Mm00434204, tumor necrosis factor α (TNF-α): Mm99999068, interferon-γ (IFN-γ): Mm99999071, transforming growth factor β (TGF-β): Mm01178820, proα2 (I) collagen (COL1A2): Mm00483888, Fibronectin 1: Mm01256744, Smad3: Mm01170760, GAPDH: Mm99999915. Real-time RT-PCR was performed on an ABI Prism 7000 sequence detector (Applied Biosystems). GAPDH was used to normalize the mRNA. The relative expression of real-time RT-PCR products was determined according to the ΔΔCt method to compare target gene and GAPDH mRNA expression, as we described previously in our study^53^.

### Statistics

All data are shown as mean ± SEM. The significance of differences between sample means was determined by the Student’s t test. Bonferroni test was used for multiple comparisons. P values less than 0.05 was considered statistically significant.

## Supplementary information

Suppementary information
