# Supplementary material for: Attenuation of murine sclerodermatous models by the selective S1P1 receptor modulator cenerimod
Source: Sci Rep. 2019 Jan 24;9:658. doi: 10.1038/s41598-018-37074-9 (PMC6345830; doi:10.1038/s41598-018-37074-9)
Supplement: Supplementary file 1 — Suppementary information [file 41598_2018_37074_MOESM1_ESM.pdf]

Supplementary Information

Attenuation of murine sclerodermatous models by the selective S1P1 receptor modulator cenerimod

Miyu Kano[1], Tadahiro Kobayashi[1], Mutsumi Date[1], Momoko Tennichi[1], Yasuhito Hamaguchi[1], Daniel S Strasser[2], Kazuhiko Takehara[1], and Takashi Matsushita[1*]

[1]Department of Dermatology, Faculty of Medicine, Institute of Medical, Pharmaceutical and Health Sciences, Kanazawa University, Kanazawa 920-8641, Japan

[2]Idorsia Pharmaceuticals Ltd., Drug Discovery, Hegenheimermattweg 91, CH-4123 Allschwil, Switzerland

Running title: Selective $S1P_1$ receptor modulator attenuates Scleroderma mouse models

[*]Address correspondence to: Takashi Matsushita, MD, PhD, Department of Dermatology, Faculty of Medicine, Institute of Medical, Pharmaceutical and Health Sciences, Kanazawa University, Kanazawa 920-8641, Japan

Phone: 81-76-265-2343 Fax: 81-76-234-4270

E-mail: t-matsushita@med.kanazawa-u.ac.jp

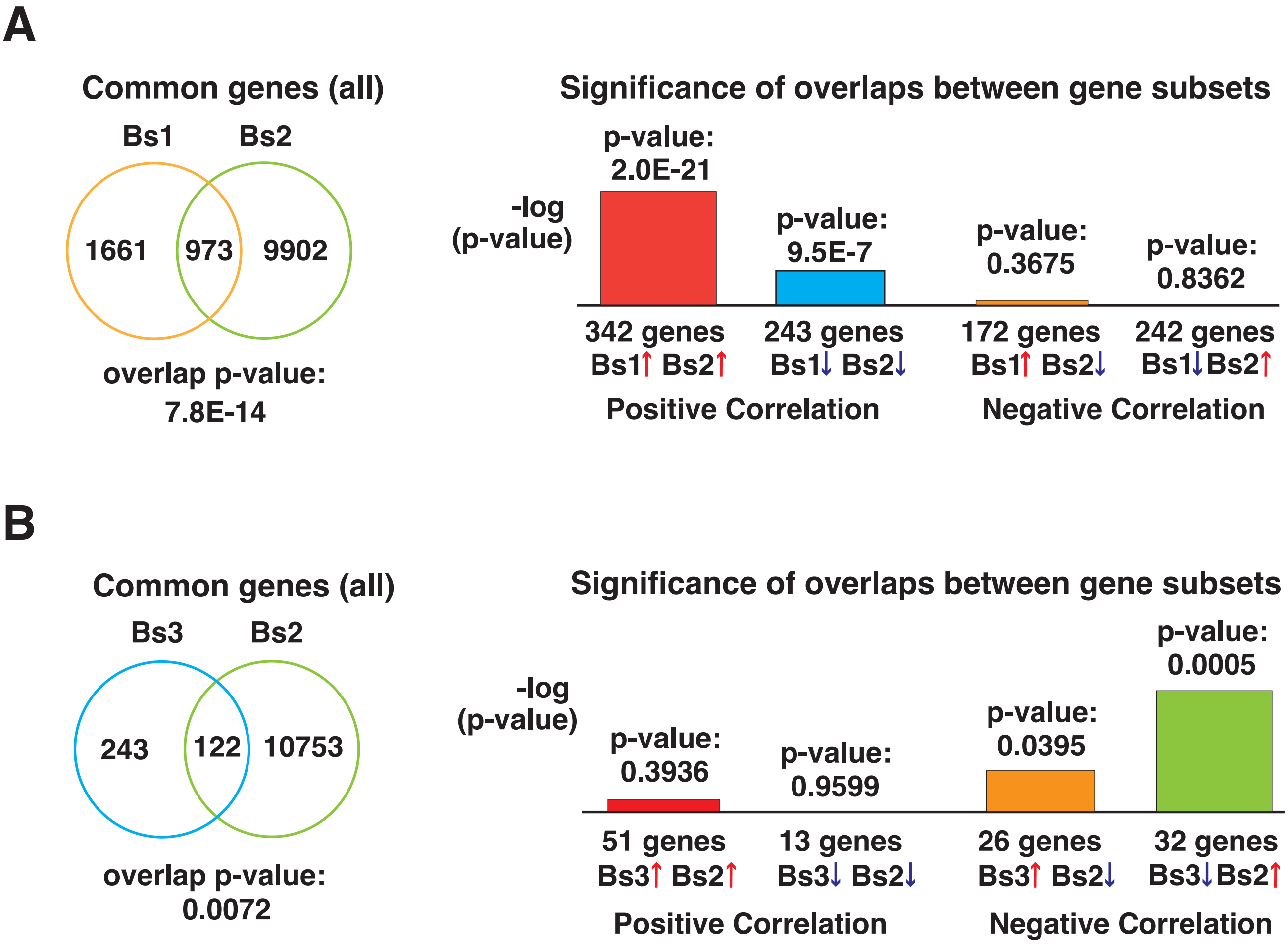

Supplementary Figure 1. (A, B) The Scl-cGVHD skin disease (Bs1) and the cenerimod (Bs3) gene expression effect was compared to a gene expression data set derived from murine Scl-cGVHD Treg cells (GSE16210 = Bs2). The analysis using Correlation Engine are showed. All data are representative of two independent experiments.
